# Inhibition of *Schistosoma mansoni* carbonic anhydrase by the antiparasitic drug clorsulon: X-ray crystallographic and *in vitro* studies

**DOI:** 10.1107/S2059798322000079

**Published:** 2022-02-18

**Authors:** Marta Ferraroni, Andrea Angeli, Simone Carradori, Claudiu T. Supuran

**Affiliations:** a Dipartimento di Chimica ‘Ugo Schiff’, University of Florence, Via della Lastruccia 3, 50019 Sesto Fiorentino, Florence, Italy; b NEUROFARBA Department, Sezione di Scienze Farmaceutiche, University of Florence, Via Ugo Schiff 6, 50019 Sesto Fiorentino, Florence, Italy; c Department of Pharmacy, ‘G. d’Annunzio’ University of Chieti-Pescara, Via dei Vestini 31, 66100 Chieti, Italy

**Keywords:** carbonic anhydrases, *Schistosoma mansoni*, clorsulon, isoform selectivity, X-ray crystallography

## Abstract

Clorsulon is an anthelmintic drug that is clinically used against *Fasciola hepatica*. Due to the presence of two sulfonamide moieties in its core nucleus, which are well recognized as zinc-binding groups, it was proposed that it may be efficacious in the inhibition of parasite carbonic anhydrases (CAs). Proteomic analyses revealed the presence of CA in the tegument of *Schistosoma mansoni*, and recently the druggability of this target was explored by testing the inhibitory activities of several sulfonamide-based derivatives. According to the principles of drug repurposing, the aim was to demonstrate a putative new mechanism of action of clorsulon and thus widen its antiparasitic spectrum. For this purpose, the inhibitory activity and isoform selectivity of clorsulon was studied using human CA I and *S. mansoni* CA, revealing different modes of binding of clorsulon that explain its inhibitory potency against the two enzymes. The information obtained in this study could be crucial in the design of more active and selective derivatives.

## Abstract 2

The inhibitory activity of clorsulon and X-ray studies of its complexes with human carbonic anhydrase I and *Schistosoma mansoni* carbonic anhydrase revealed different modes of binding of this antiparasitic drug, explaining its inhibitory potency against the two enzymes.

## 1. Introduction

**Figure 1:**
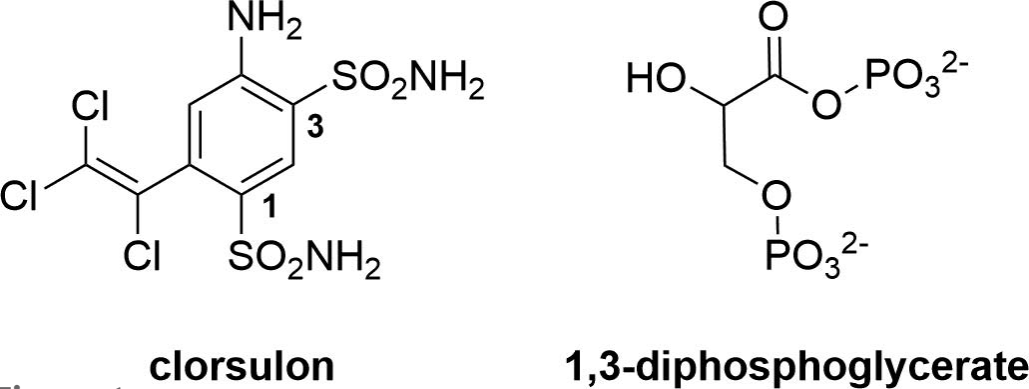
Structures of clorsulon and 1,3-diphosphoglycerate.

4-Amino-6-(1,2,2-trichlorovinyl)benzene-1,3-disulfonamide, commercially known as clorsulon, is a 1,3-disulfonamide that is structurally similar to 1,3-diphosphoglycerate (Fig. 1 ▸) and is able to selectively inhibit parasitic magnesium-containing enzymes implicated in the glycolytic pathway, namely phosphoglycerate kinase (EC 2.7.2.3; PGK 1) and phosphoglyceromutase (EC 5.4.2.11; PGM) (Schulman *et al.*, 1982 ▸). As a competitive inhibitor, it blocks the oxidation of glucose to acetate and propionate (the Embden–Meyerhoff pathway), thus limiting the primary source of energy in flukes under anaerobic conditions (Martin, 1997 ▸; Timson, 2016 ▸). It is approved for veterinary use in cattle against adult liver flukes such as *Fasciola hepatica* and *F. gigantica* as an oral suspension and by subcutaneous injection (Meaney *et al.*, 2003 ▸).

Clorsulon has been shown to possess a wider antiparasitic activity in various animal models of disease, in which its pharmacokinetics and toxicity were also assessed. In particular, an *in vivo* study reported a weak carbonic anhydrase (CA) inhibitory activity in rats (Lankas & Peter, 1992 ▸), which was also suggested by *SwissTargetPrediction* (Daina *et al.*, 2019 ▸). This commercially available tool reported that many human CAs may putatively be inhibited by this drug.

Carbonic anhydrases (EC 4.2.1.1) are widespread metallo­enzymes that can be clustered into eight families according to their amino-acid characteristics and differences in their active centers. They are present in all living organisms, catalyzing the hydration of carbon dioxide to bicarbonate and a proton (Supuran, 2016*a* ▸). Despite the presence of α-CAs in both vertebrates and helminths, enzymatic and X-ray crystallo­graphic studies have pinpointed differences in their kinetics and inhibition (Zolfaghari Emameh *et al.*, 2016 ▸). *F. hepatica* displayed various membrane-associated enzymes/proteins in its tegument (including carbonic anhydrase), which must play important roles in host–parasite communication, immune-system evasion strategies and metabolite transport and transformation (Wilson *et al.*, 2011 ▸). Orthologues of several proteins identified in the transcriptome of *F. hepatica* have previously been reported in the tegument of *Schistosoma mansoni* (Braschi *et al.*, 2006 ▸; Castro-Borges *et al.*, 2011 ▸).

*S. mansoni* is the causative agent of schistosomiasis, a neglected tropical disease that primarily affects the poorest populations worldwide. Along with the other five *Schistosoma* species, *S. mansoni* is characterized by a complex life cycle involving asexual multiplication in an intermediate host and a sexual phase in mammals. The most used drug is praziquantel, which is endowed with a broad antiparasitic spectrum, well known pharmacokinetics and tolerability. Its mechanism of action is directed towards increasing the influx of Ca^2+^ across the tegument and the harmful contraction of the parasite.

Conversely, its massive use has led to the development of improved resistance, with no alternative options being proposed in recent years (McManus *et al.*, 2018 ▸; Wilson, 2020 ▸; Siqueira *et al.*, 2017 ▸).

In contrast to that from *F. hepatica*, *S. mansoni* carbonic anhydrase (SmCA) has recently and elegantly been exploited as an attractive target for the design of new and innovative parasite inhibitors. It is expressed on the surface of the schistosome, and suppression of its activity by RNAi significantly halted larval infectivity in mice. Further information was extrapolated by kinetic experiments and X-ray crystallo­graphy, unraveling unique structural characteristics for the development of inhibitors (Da’dara *et al.*, 2019 ▸). Like human α-CAs, SmCA can be efficiently targeted by (hetero)aryl­sulfonamide-type chemical scaffolds belonging to newly synthesized compounds or to repurposed drugs (Angeli *et al.*, 2020 ▸, 2021 ▸). Sulfonamides and their bioisosteres are the main inhibitors of CA, with inhibition constants in the nanomolar range, because of the presence of an undoubtedly efficacious zinc-binding group (ZBG; Biswas *et al.*, 2011 ▸; Mishra *et al.*, 2020 ▸; Akgul *et al.*, 2021 ▸), although an increased isoform selectivity must be achieved by the introduction of additional moieties on the aryl ring, as in the tail approach.

Collectively, the presence of two sulfonamide groups on the aryl ring of clorsulon along with its preliminary effect on rat CA *in vivo* prompted us to further investigate its inhibitory profiles against human and parasite CAs. Moreover, to assess selectivity we also performed experiments using a large panel of different representatives from the CA families. *In vitro* enzymatic tests and X-ray experiments will provide the basis for the putative application of this drug to other parasitic diseases, deciphering a new mechanism of action. The results of this study may undoubtedly be useful to enlarge the therapeutic arsenal available against schistosomiasis and propose clorsulon as an alternative option or in combination with praziquantel, which is characterized by a different mechanism of action.

## 2. Methods and materials

### 2.1. Chemicals

Clorsulon (purity ≥98%) and acetazolamide (purity ≥99%) were supplied by Sigma–Aldrich, Milan, Italy. All other reagents were of analytical grade.

### 2.2. Evaluation of CA-inhibitory activity

An Applied Photophysics stopped-flow instrument was used to assay the CA-catalysed CO_2_-hydration activity. Phenol red (at a concentration of 0.2 m*M*) was used as an indicator, working at an absorbance maximum of 557 nm, with 20 m*M* HEPES pH 7.5 or Tris pH 8.3 (for β-CAs, γ-CAs and δ-CAs) as buffer and 20 m*M* sodium sulfate (to maintain a constant ionic strength), following the initial rates of the CA-catalyzed CO_2_-hydration reaction for a period of 10–100 s. The CO_2_ concentrations ranged from 1.7 to 17 m*M* for determination of the kinetic parameters and inhibition constants. For each inhibitor, at least six traces of the initial 5–10% of the reaction were used to determine the initial velocity. The uncatalyzed rates were determined in the same manner and were subtracted from the total observed rates. Stock solutions of inhibitor (0.1 m*M*) were prepared in distilled deionized water and dilutions of up to 0.01 n*M* were made in the assay buffer. Inhibitor and enzyme solutions were pre-incubated together for 15 min at room temperature prior to the assay in order to allow formation of the enzyme–inhibitor complex. The inhibition constants were obtained by nonlinear least-squares methods using *Prism* 3 and the Cheng–Prusoff equation, as reported previously, and represent the mean from at least three different determinations. All CA isoforms were recombinant isoforms obtained in-house, as reported previously (De Vita *et al.*, 2017 ▸; Angeli *et al.*, 2017 ▸, 2019 ▸).

### 2.3. Expression and purification of recombinant SmCA

The full-length coding sequence of SmCA (GenBank accession No. MK611932), including the predicted signal peptide and GPI-anchor domain, was codon-optimized using hamster codon preferences and synthesized commercially (GenScript). Next, the region encoding amino acids Asn21–Ala298 (*i.e.* lacking the amino-terminal signal peptide and the carboxyl-terminal GPI-anchoring signal) was generated by PCR using forward and reverse primers containing AscI and XhoI restriction sites, respectively, with the synthetic codon-optimized gene as a template. The amplified product was cloned into the pSecTag2A plasmid (Invitrogen) at the AscI and XhoI sites in-frame with the Igκ leader sequence at the 5′-end and a Myc epitope and a six-histidine tag at the 3′-end. Successful in-frame cloning was confirmed by sequencing at the Tufts University Core Facility.

To express recombinant SmCA (rSmCA), suspension-adapted FreeStyle Chinese hamster ovary (CHO-S) cells were transfected with the plasmid using FreeStyle MAX Reagent following the manufacturer’s instructions (Invitrogen). Cells were harvested at various time points post-transfection to monitor the viability (using a trypan blue exclusion test) and the expression of rSmCA (using Western blotting). To facilitate protein production, stable cell-line clones secreting rSmCA were selected by treating transfected cells with 250 µg ml^−1^ Zeocin for two weeks; individual clones that produced high yields (5–10 mg) of purified active rSmCA per litre were maintained.

Recombinant SmCA was purified from the cell-culture medium by standard immobilized metal-affinity chromatography (IMAC) using HisTrap Excel columns (GE Healthcare Life Sciences) following the manufacturer’s instructions. Purified recombinant protein eluted from the column was dialyzed overnight at 4°C against 50 m*M* Tris–HCl pH 7.4, 150 m*M* NaCl and then concentrated by centrifugal ultrafiltration (Pierce Protein Concentrators, 10K MWCO, Thermo Scientific). The final protein concentration was determined using a BCA Protein Assay Kit (Pierce). Aliquots of eluted protein were resolved by 4–20% SDS–PAGE to assess their purity and some were tested for specificity by Western blotting using anti-Myc tag and anti-SmCA antibodies.

### 2.4. Crystallization and X-ray data collection

The SmCA enzyme was crystallized at 296 K using the sitting-drop vapor-diffusion method in 96-well plates (CrystalQuick, Greiner Bio-One, Germany). Drops were prepared by mixing 1 µl protein solution with 1 µl reservoir solution and were equilibrated against 100 µl precipitant solution. The concentration of the protein was 10 mg ml^−1^ in 50 m*M* Tris pH 8.3. The initial crystallization conditions were found using the JCSG-*plus* screen (Molecular Dimensions) and were optimized. Crystals of SmCA were obtained using 20% PEG 6000, 0.1 *M* citrate pH 5.0. The crystals belonged to the primitive trigonal space group *P*3_2_21. The complex was prepared by soaking native SmCA crystals in mother-liquor solution containing the inhibitor at a concentration of 10 m*M* for two days. Crystals of hCA I were obtained by the hanging-drop vapor-diffusion method using a 24-well Linbro plate. 2 µl of a 10 mg ml^−1^ solution of hCA I in 20 m*M* Tris–HCl pH 9.0 were mixed with 2 µl of a solution of 28–31% PEG 4000, 0.2 *M* sodium acetate, 0.1 *M* Tris pH 8.5–9.0 and were equilibrated against the same solution at 296 K. The complex was prepared by soaking the native hCA I crystals in mother-liquor solution containing the inhibitor at a concentration of 10 m*M* for two days. All crystals were flash-cooled at 100 K using a solution obtained by adding 15%(*v*/*v*) glycerol to the mother-liquor solution as a cryoprotectant. Data were collected from crystals of the complexes using synchrotron radiation on the XRD2 beamline at Elettra Synchrotron, Trieste, Italy with a wavelength of 0.971 Å and a Dectris PILATUS 6M detector. The data were integrated and scaled using *XDS* (Leslie & Powell, 2007 ▸). Data-processing statistics are shown in Table 1 ▸.

### 2.5. Structure determination

The crystal structures of hCA I (PDB entry 1jv0; Ferraroni *et al.*, 2002 ▸) and SmCA (PDB entry 6qqm; Da’dara *et al.*, 2019 ▸) without solvent molecules and other heteroatoms were used to obtain the initial phases using *REFMAC*5 (Murshudov *et al.*, 2011 ▸).

5% of the unique reflections were randomly selected and excluded from the refinement data set for the purpose of *R*_free_ calculations. The initial |*F*_o_ − *F*_c_| difference electron-density maps unambiguously showed the inhibitor molecules. The inhibitor was introduced into the model with an occupancy of 1.0 or 0.7. Refinement proceeded using the normal protocols of positional, isotropic atomic displacement parameters alternating with manual building of the models using *Coot* (Emsley *et al.*, 2010 ▸). The quality of the final models was assessed with *Coot* and *RAMPAGE* (Lovell *et al.*, 2003 ▸). Crystal parameters and refinement data are summarized in Table 1 ▸. The atomic coordinates have been deposited in the Protein Data Bank (PDB entries 7plf and 7pri). Graphical representations were generated with *UCSF Chimera* (Pettersen *et al.*, 2004 ▸).

## 3. Results and discussion

Like other sulfonamides, both clorsulon and acetazolamide (as a reference drug) markedly interacted with all of the selected isoforms belonging to different CA families, as reported in Table 2 ▸. Human CAs were better inhibited by AAZ, whereas clorsulon displayed an inferior affinity for hCA I (*K*_i_ = 4516 n*M*) with respect to hCA II, IX and XII. On the other hand, SmCA from *S. mansoni* was strongly affected by clorsulon (*K*_i_ = 224.7 n*M*) and AAZ (*K*_i_ = 42.5 n*M*), despite the latter being more potent. The selectivity index (SI) of clorsulon was about fourfold better than that of AAZ for human CA I. As reported previously, AAZ also inhibited the two *Caenorhabditis elegans* β-CAs, but it suffered from poor penetration into the nematode cuticle (Hall *et al.*, 2008 ▸). With regard to other α-CAs (SazCA from *Sulfurihydrogenibium azorense* and VhCAα from *Vibrio cholerae*), clorsulon acted very efficaciously, in particular against SazCA, with a *K*_i_ of 7.7 n*M* and a selectivity index (SI) of 580-fold with respect to hCA I. This selectivity can also be found, although to a lesser extent, against VhCAα and indicates the possibility of employing clorsulon against other pathogenic microorganisms. Clorsulon showed less potency towards β-CAs (VhCAβ from *V. cholerae*, BpsCAβ from *Burkholderia pseudomallei* and mtCA 03 from *Mycobacterium tuberculosis*), with the only exception being mtCA 02. BpsCAγ from *B. pseudomallei* belonging to the γ-CA family was almost unaffected, whereas a δ-CA, represented by TweCA from the marine diatom *Thalassiosira weissflogii*, was inhibited by nanomolar concentrations of clorsulon.

The inhibitory differences of clorsulon, in terms of structural changes, were studied by X-ray diffraction experiments using hCA I (considered to be the least-inhibited human off-target) and SmCA. Detailed data-collection and refinement statistics are reported in Table 1 ▸.

**Figure 2:**
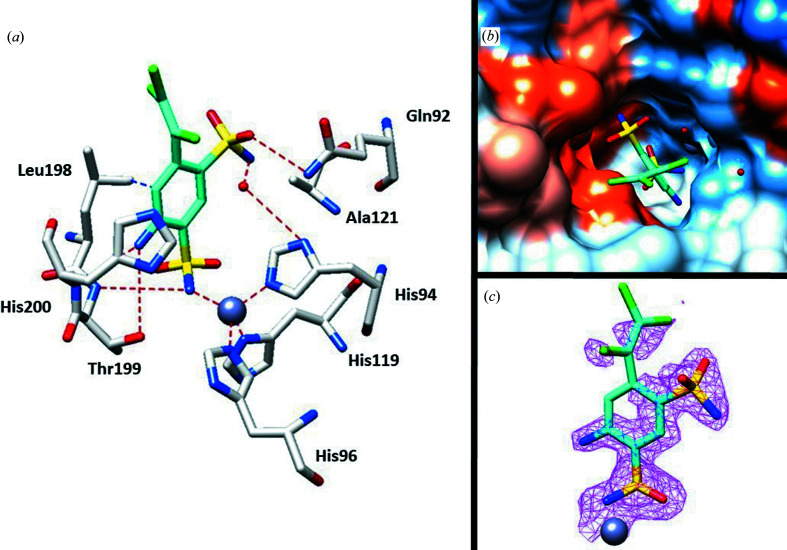
(*a*) X-ray crystal structure of clorsulon bound to hCA I (PDB entry 7plf). (*b*) Clorsulon inside the active site of hCA I. Hydrophobic (red) and hydrophilic (blue) residues are labeled. (*c*) 2*F*_o_ − *F*_c_ electron-density map of clorsulon bound to zinc in the hCA I active site contoured at the 1.0σ level. Residues involved in the binding of inhibitors are also shown; the gray sphere represents the Zn atom in the active site of the protein.

After the initial rounds of refinement of the complex of clorsulon and hCA I, the calculated *F*_o_ − *F*_c_ map showed clear electron density inside the active site compatible with the inhibitor. Cl atoms were introduced into the model with zero occupancy due to the fact that there was scarce electron density around this portion of the molecule (Fig. 2 ▸ *c*). Clorsulon contains two sulfonamide groups and it is interesting to note that the sulfonamide which interacts directly with the zinc ion is that in position 3. This choice was probably due to steric hindrance by the trichlorovinyl moiety in position 6. In addition, this portion formed the characteristic hydrogen bond to residue Thr199 that stabilizes the interaction typical of this class of inhibitors (Fig. 2 ▸; Pinard *et al.*, 2015 ▸; Supuran, 2016*b* ▸; Tanini *et al.*, 2021 ▸).

The amino group in position 4 formed two hydrogen bonds to the side chains of Thr199 and His200, whereas the second sulfonamide moiety interacted with the side chain of Gln92 and established a water bridge to His94. On the other hand, regarding the hydrophobic interactions, the aromatic ring of clorsulon showed a connection to Leu198.

**Figure 3:**
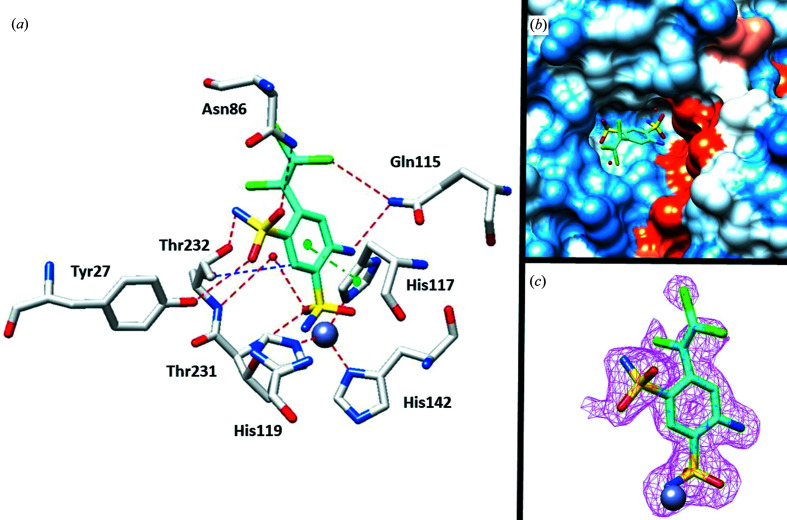
(*a*) X-ray crystal structure of clorsulon bound to SmCA (PDB entry 7pri). (*b*) Clorsulon inside the active site of SmCA. Hydrophobic (red) and hydrophilic (blue) residues are labeled. (*c*) 2*F*_o_ − *F*_c_ electron-density map of clorsulon bound to zinc in the SmCA active site contoured at the 1.0σ level. Residues involved in the binding of inhibitors are also shown and the gray sphere represents the Zn atom in the active site of the protein.

In the clorsulon–SmCA complex (Fig. 3 ▸) clear electron density for the compound was again observed inside the active site of the protein (Fig. 3 ▸ *c*). The benzenesulfonamide scaffold showed the same binding mode as mentioned above, with the hydrogen bond between the O atom of the sulfonamide group and Thr231 stabilizing the binding of the inhibitor, as in the case of the complex with hCA I.

In addition, this moiety formed a water bridge to Thr232. The second sulfonamide moiety played an important role in the location of the inhibitor; indeed, three hydrogen bonds were observed to Thr232, Tyr27 and Asn86, blocking the scaffold in a hydrophilic pocket inside the active site (Fig. 3 ▸ *a*). This location offers the inhibitor the possibility of forming a π-stacking interaction with the aromatic ring of His117, which is absent in the complex with the human isoform. Finally, the amino group formed a hydrogen bond to Gln115 (Fig. 3 ▸).

**Figure 4:**
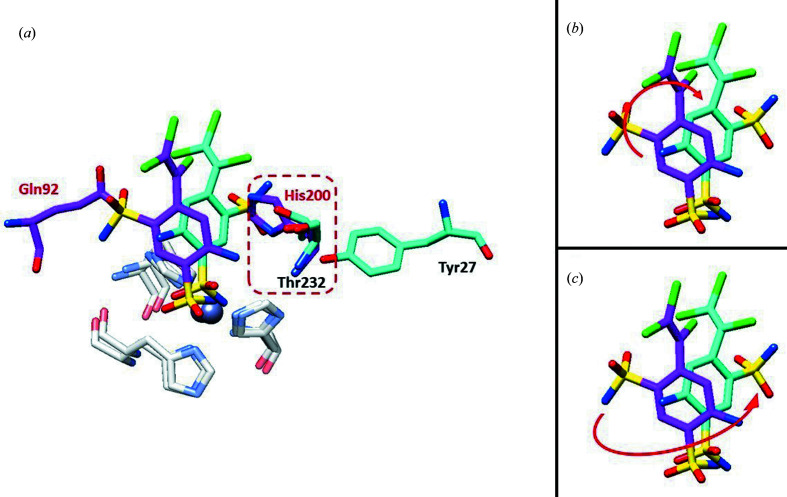
(*a*) Overlay of clorsulon with hCA I (magenta) and SmCA (cyan). Specific residues are labeled in red for hCA I and in cyan for SmCA. (*b*) Benzene-ring rotation of clorsulon with hCA I (magenta) and SmCA (cyan). (*c*) Sulfonamide-group rotation of clorsulon with hCA I (magenta) and SmCA (cyan).

Although a structural comparison of clorsulon bound to hCA I and SmCA showed similar features, such as the same sulfonamide moiety interacting with the catalytic zinc ion and the hydrogen bond to Thr199 or Thr231 (in hCA I and SmCA, respectively), the remaining part of the molecule showed substantial differences, as outlined in Fig. 4 ▸. These differences could explain the different potency of inhibition against hCA I and SmCA (*K*_i_ values of 4516 and 224.7 n*M*, respectively).

A superposition of the two structures clearly shows how the different side chain at the position of His200 in hCA I/Thr232 in SmCA plays a key role in the orientation of the inhibitor (Fig. 4 ▸ *a*). Indeed, His200 in hCA I reduces the space available for the clorsulon tail and forces the second sulfonamide moiety (which is bulkier than the amino group) to move towards Gln92 (Gln115 in SmCA). On the other hand, in SmCA this residue is replaced by Thr232 with a less bulky side chain, which makes the active site more accessible, driving the sulfonamide moiety towards a more hydrophilic pocket, with a rotation of 130° with respect to the hCA I complex (Fig. 4 ▸ *c*), and also allowing the formation of interactions with Tyr27 and Asn86 (Tyr7 and Val62 in hCA I). In addition, a consequent benzene-ring rotation of 35° (Fig. 4 ▸ *b*) results in the formation of a π-stacking interaction with His117 (His94 in hCA I) that is absent in the hCA I complex. Taken together, these observations could explain the different inhibition potencies of clorsulon against hCA I and SmCA and the 20-fold selectivity for SmCA over hCA I.

## 4. Conclusions

*S. mansoni* causes a neglected tropical disease in many developing countries and affects low-income rural communities. The preventive and therapeutic mainstay is praziquantel, a safe and efficacious drug that is orally active against adult schistosoma worms. Reinfections, resistance and a lack of vaccines make the discovery of an alternative drug the only option beyond snail control and sanitation measures. Keeping in mind the presence of carbonic anhydrase in the tegument of this parasite and knowing that sulfonamide-type compounds are able to strongly inhibit this enzyme, we aimed to explore the inhibitory activity of clorsulon, a drug that is currently used against another parasite (*F. hepatica*).

Clorsulon inhibited SmCA at nanomolar concentrations (*K*_i_ of 224.7 n*M*), displaying selectivity over the human CA I isoform (SI = 20.09). The inhibitory profile of this bisulfonamide against CAs belonging to different families was assessed and in-depth X-ray crystallography of clorsulon in complex with hCA I and SmCA revealed structural differences which can be used to obtain a better safety profile or for the design of more active derivatives. The high inhibition of hCA II could be responsible for side effects resulting from the use of clorsulon in humans, so that clarifying the interactions of the drug with hCA II will be of the utmost importance. Moreover, further studies are ongoing to corroborate these data in *in vivo* murine models of infection with *S. mansoni*.

## Supplementary Material

PDB reference: human carbonic anhydrase I, complex with clorsulon, 7plf

PDB reference: carbonic anhydrase from *Schistosoma mansoni*, complex with clorsulon, 7pri

## Figures and Tables

**Table 1:** Summary of data-collection and atomic model-refinement statistics for hCA I and SmCA

	hCA I + clorsulon	SmCA + clorsulon
PDB code	7plf	7pri
Data-collection statistics		
Wavelength (Å)	0.971800	0.971900
Space group	*P*2_1_2_1_2_1_	*P*3_2_21
*a*, *b*, *c* (Å)	62.27, 71.34, 121.41	103.80, 103.80, 132.90
α, β, γ (°)	90.00, 90.00, 90.00	90.00, 90.00, 120.00
Resolution (Å)	46.92–1.46 (1.50–1.46)	48.00–1.68 (1.72–1.68)
Unique reflections	94318 (6929)	94460 (6740)
*R*_merge_ (%)	6.3 (119.1)	10.1 (132.3)
*R*_meas_ (%)	6.5 (124.1)	9.9 (125.3)
Multiplicity	12.46 (12.63)	17.26 (8.93)
Overall completeness (%)	100.0 (100.0)	99.7 (97.2)
〈*I*/σ(*I*)〉	20.00 (2.25)	27.43 (2.32)
CC_1/2_	99.9 (83.8)	100.0 (70.6)
Refinement statistics		
Resolution range (Å)	46.957–1.461	89.90–1.68
*R* factor (%)	17.72 (27)	16.69 (26)
*R*_free_ (%)	20.50 (28)	19.51 (26)
R.m.s.d., bond lengths (Å)	0.0130	0.0128
R.m.s.d., angles (°)	1.8247	1.8318
Ramachandran statistics (%)		
Most favored	97.1	97.1
Additionally allowed	2.9	2.9
Outlier regions	0.0	0.0
Average *B* factors (Å^2^)		
All atoms	24.031	24.590
Inhibitors	44.456	38.721
Solvent	31.741	29.464

**Table 2:** *In vitro* inhibitory activity and selectivity index (SI) of clorsulon and acetazolamide (AAZ) as a reference sulfonamide-based drug against human CAs (hCA I, hCA II, hCA IX and hCA XII), schistosomal CA (SmCA) and other CAs belonging to different families, as assessed by a stopped-flow CO_2_-hydration assay (Khalifah, 1971 ▸)

		*K*_i_ † (n*M*)	
		Clorsulon	AAZ
α-CAs	hCA I	4516	250.0
	hCA II	59.1	12.1
	hCA IX	137.5	25.7
	hCA XII	78.0	5.7
	SmCA	224.7	42.5
	VhCAα	291.7	6.8
	SazCA	7.7	0.9
β-CAs	VhCAβ	906.1	6.8
	BpsCAβ	223.3	745.0
	mtCA 02	2.9	9.8
	mtCA 03	827.5	104.0
γ-CA	BpsCAγ	2500	149.0
δ-CA	TweCA	95.8	83.0
SI (hCA I/SmCA)		20.09	5.88

† Mean from three different assays using a stopped-flow technique (errors were in the range ±5–10% of the reported values).
